# 5-Azacytidine increases tanshinone production in *Salvia miltiorrhiza* hairy roots through epigenetic modulation

**DOI:** 10.1038/s41598-022-12577-8

**Published:** 2022-06-07

**Authors:** Bo-Cheng Yang, Meng-Shiou Lee, Ming-Kuem Lin, Wen-Te Chang

**Affiliations:** Department of Chinese Pharmaceutical Sciences and Chinese Medicine Resources, College of Chinese Medicine, China Medical University, Taichung, Taiwan

**Keywords:** Plant biotechnology, Secondary metabolism

## Abstract

Recent studies have indicated strong connections between epigenetic modulation and secondary metabolites in plants. It is vital to understand the roles of epigenetics in the production of secondary metabolites. In this study, the inhibitor of DNA methylation 5-azacytidine (5-Az) was used on the hairy roots of the medicinal plant *Salvia miltiorrhiza* to investigate its effect on secondary metabolite production, gene expression, methylation levels in genomic DNA and promoter regions. Our results showed that the contents of tanshinones in *S. miltiorrhiza* hairy roots increased by 1.5–5 times, and some genes in the biosynthesis pathway showed an upward trend. According to our NGS analysis, the methylation pattern in the promotor of the gene encoding copalyl diphosphate synthase (CPS) was altered, and 51 out of 145 cytosines were demethylated during 5-Az treatment. A total of 36 putative transcription factors (TFs) binding cites were identified in these demethylation sites. Among these TFs binding cites, cis-regulatory elements for the binding of NF-Y and MYB were frequently found in our results. This is the first report to demonstrate a possible mechanism of DNA methylation participating in tanshinone biosynthesis in *S. miltiorrhiza* hairy roots by modulating the *CPS* promoter and TFs binding sites.

## Introduction

Epigenetics is a branch of genetics proposed by Conrad Waddington in 1942^1^. It investigates how cells can achieve reversible and/or heritable changes in gene expression without DNA sequence alterations to ensure survival upon environmental fluctuations^2,3^. The underlying mechanisms of epigenetic regulation include DNA methylation, histone modifications, chromatin remodeling, and noncoding RNA (ncRNA)-mediated regulation of gene expression. Among them, DNA methylation is that the methyl group of *S*-adenosylmethionine (SAM) is transferred to the carbon 5 of cytosine at CG, CNG and CNN (where N could be any nucleotide except G) sequences in DNA. The content of 5-methylcytosines is increased through the catalysis of DNA methyltransferase (DNMT) in genomic DNA^4^. In higher plants, several studies have reported that various environmental factors can cause alterations in DNA methylation levels. Changes in methylation patterns in promoter regions might regulate gene expression related to stress resistance^5,6^. For instance, wheat cultivars with different salinity tolerances showed dissimilar methylation patterns and gene expression results when exposed to salinity stress. Demethylation in the promoter region affected the expression of stress resistance genes and improved their survival during environmental fluctuations. This result suggested that DNA methylation might be a key mechanism in the regulation of salinity tolerance in wheat^7,8^.

Since environmental stresses have large impacts on plants, some unique strategies, such as stress escape and/or stress tolerance, have been developed to lower the negative effects on their life cycle^9,10^. Plant secondary metabolites are one of the strategies when plants are exposed to abiotic/biotic stresses, such as water shortages, insect attacks, light, and drought^11^. Secondary metabolites might act as mediators to survive in a tough situation, and their production might be modulated through epigenetic regulation^12,13^. Recent studies have indicated strong connections between DNA methylation and the production of secondary metabolites, but the relationships have not yet been clearly investigated^14,15^. Possible mechanisms have been proposed that DNA methylation could affect the chromatin structure and lead to gene modulation^16^. Some studies also reported that DNA methylation could affect the transcription factors (TFs) binding and resulted in gene regulation^17^. TFs are proteins that could bind to DNA and regulate gene expression, while some of them might act as a stress-responsive factor under environmental challenges^18^. Serval studies have indicated that the expression of certain TFs might improve the stress tolerance and modulate the production of secondary metabolites^19–22^.

Plant hairy root is a secondary metabolite research platform with the characteristics of rapid growth, easy maintenance, and stable genetic and biochemical characteristics. Plant hairy roots can be induced by *Agrobacterium rhizogenes* infection to investigate secondary metabolite production and their biosynthesis pathways^23^. In recent years, the study of secondary metabolite production by *Salvia miltiorrhiza* hairy roots through the elicitation of physical and chemical treatments has been widely investigated with excellent results^24–27^. *S. miltiorrhiza* (also called Danshen in Chinese, belonging to the Lamiaceae family) is a famous medicinal plant worldwide and it is extensively used for gynecology in traditional Chinese medicine (TCM). It has multiple functions, such as invigorating blood, nourishing blood, relieving menstruation and relieving pain. Two main type of components from *S. miltiorrhiza* have been reported to play key roles for these functions. One is water-soluble phenolic acid compounds, such as rosmarinic acid and salvianolic acid B. Two biosynthesis pathways are involved in the production of hydrophilic phenolic acids, namely the phenylpropanoid pathway and tyrosine-derived pathway^28,29^. Current studies showed that enzymes, such as 4-hydroxyphenylpyruvate reductase (HPPR) and tyrosine aminotransferase (TAT), in the tyrosine-derived pathway were more correlated to phenolic acid production, and they implied that the tyrosine-derived pathway might be the rate-limiting step in biosynthesis of phenolic acids^30–32^. The other is liposoluble tanshinone compounds, such as tanshinone IIA, tanshinone I, dihydrotanshinone and cryptotanshinone, that protect the myocardium and have anticancer effects^33,34^*.* The biosynthetic pathway of tanshinones can be divided into 3 steps: the production of terpenoid precursors, the construction of tanshinones skeletons, and modification of skeletons. Terpenoid precursors can be produced through two biosynthetic pathways, namely the mevalonate (MVA) pathway in the cytosol and the methylerythritol phosphate (MEP) pathway in plastids. To prove their critical role on synthesis pathway, most genes in both pathways have been cloned in *S. miltiorrhiza*. Several reports have revealed that tanshinones were mainly synthesized on MEP pathway instead of MVA pathway^35,36^. Then, those precursors are catalyzed into the skeleton of tanshinones, and eventually other structural modification might happen later on the P450s^37,38^. It is noteworthy that the terpenoid synthases are the key enzymes for the biosynthesis of tanshinone skeletons. Copalyl diphosphate synthase (CPS) and kaurene synthase-like (KSL) are involved in forming the skeleton miltiradiene. RNA interference was applied to decreased the *CPS* expression in *S. miltiorrhiza* hairy roots, and caused significant drops in the content of tanshinones^39^. These results revealed the vital character of *CPS* in tanshinone biosynthesis.

5-Azacytidine (5-Az) is a nucleotide analog that is used as a DNA methyltransferase inhibitor. 5-Az can selectively activate gene expression in eukaryotic cells and change the state of cell differentiation in specific cells^40,41^. 5-Az was originally developed and tested as a nucleoside antimetabolite for acute myelogenous leukemia in humans. It also has antibacterial, antitumor, suppresses immunity, inhibits mitosis, protects against radiation, and inhibits virus replication^42,43^. The mechanism of 5-Az is randomly incorporated into newly synthesized DNA strands where it irreversibly binds to DNA methyltransferases and then decreases DNA methyltransferase activity. Eventually, the genome would result in hypomethylation at random sequences^44,45^. 5-Az could induce various plant phenotype changes, including dwarfism, early flowering and inhibition of vegetative growth^46^. In addition, the stimulating effect of DNA methyltransferase inhibitors on the accumulation of secondary metabolites has also been investigated in cell suspension cultures of *Catharanthus roseus*, *Betula platyphylla* and *Vitis amurensis*^47–49^.

In our study, the 5-Az was used on the hairy root system of *S. miltiorrhiza* to systematically investigate the effects on secondary metabolite production via regulation of DNA methylation. The gene expression of key enzymes for phenolic acid and tanshinone compound biosynthesis in *S. miltiorrhiza* hairy roots was also addressed using sensitive qualitative and quantitative techniques. In terms of the alternation in DNA methylation pattern, the level of DNA methylation in genomic DNA and the *CPS* promoter were also investigated. This is the first report to simultaneously demonstrate and compare phenolic acid and tanshinone compound biosynthesis in *S. miltiorrhiza* hairy roots under epigenetic modulation.

## Results

### Growth and morphology were affected by various 5-Az concentrations

**Figure 1:**
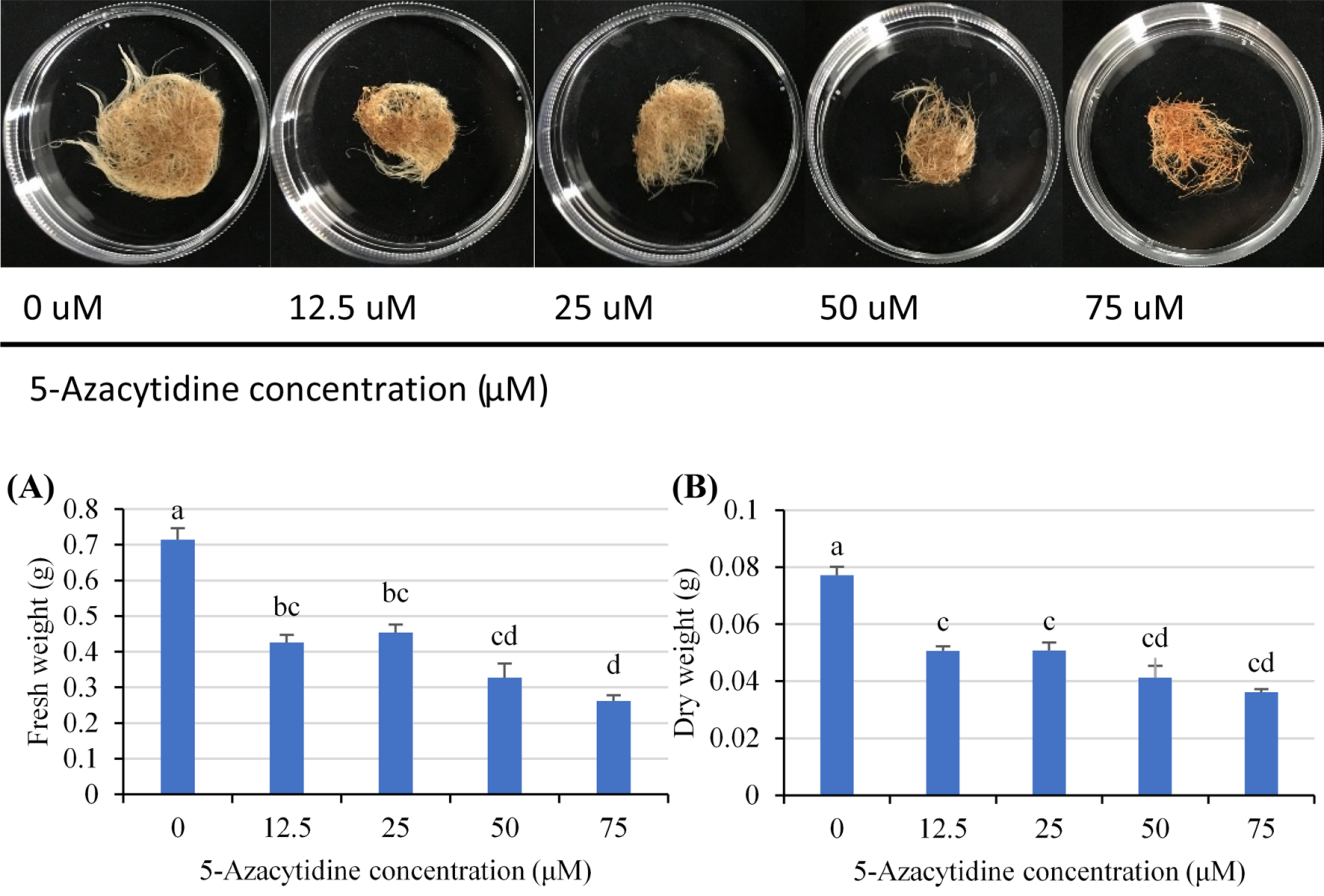
Influence of different concentrations of 5-azacytidine on *Salvia miltiorrhiza* hairy roots (**A**) fresh weight and (**B**) dry weight after 28 days of treatment. The values are the mean of at least three replicates ± S.E. Different letters indicate significant differences at the 5% level according to the LSD test.

Growth and morphological changes in *S. miltiorrhiza* hairy roots with different concentrations of 5-Az are shown in Fig. 1. The fresh weight and dry weight were measured after 28 days of treatment, and significant growth retardation of hairy roots was shown with 5-Az treatment. The fresh weight and the dry weight of hairy roots decreased by approximately 40% when the medium contained 12.5 and 25 μM 5-Az. The mass of hairy roots decreased by more than half when the medium contained greater than 50 μM 5-Az. Besides, the notable red color changes can be observed when hairy roots treated with 5-Az, especially in the treatment with high concentration of 5-Az. It might be caused by the accumulation of tanshinones components. Meanwhile, the end of the hairy roots also swelled a bit up compared with control group after 28 days of treatment.

### Liposoluble active component production was increased significantly

**Figure 2:**
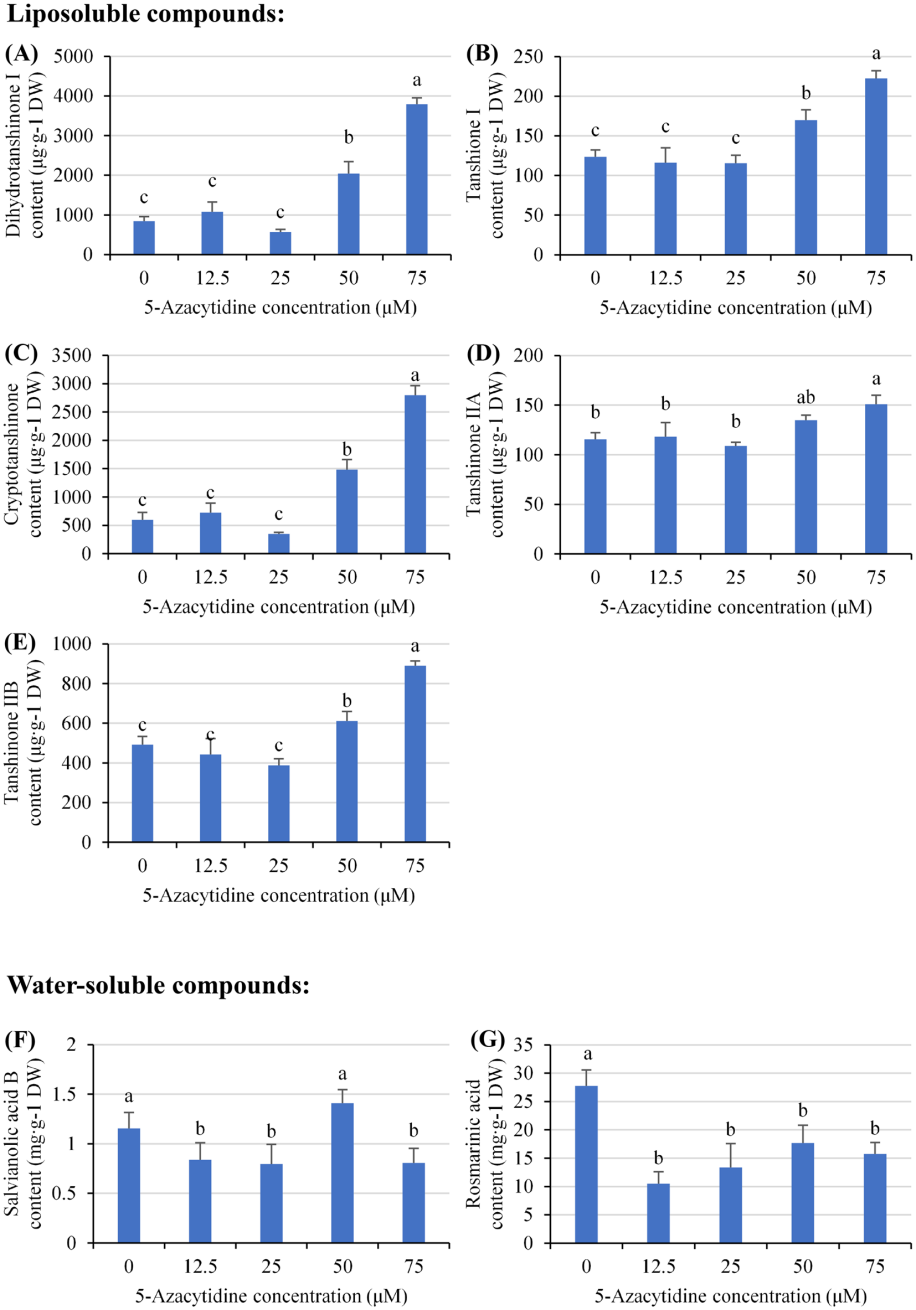
Contents of (**A**) dihydrotanshinone I, (**B**) tanshinone I, (**C**) cryptotanshinone, (**D**) tanshinone IIA, (**E**) tanshinone IIB, (**F**) rosmarinic acid, and (**G**) salvianolic acid B under different levels of 5-azacytidine in *Salvia miltiorrhiza* hairy root cultures after 28 days. The values are the mean of at least three replicates ± S.E. Different letters indicate significant differences at the 5% level according to the LSD test.

The active components in *S. miltiorrhiza* hairy roots were measured after treatment with different concentrations of 5-Az for 28 days. As illustrated in Fig. 2, the amounts of liposoluble components were not affected when the concentration of 5-Az was below 25 μM. In particular, the production of liposoluble components, including dihydrotanshinone I, tanshinone I, cryptotanshinone, tanshinone IIA and tanshinone IIB, was increased by 1.5–5 times compared with that of the control groups when the 5-Az concentration was greater than 50 μM (Fig. 2A–E). In contrast to the liposoluble components, 5-Az decreased the production of water-soluble components in hairy roots. Exception of the content of salvianolic acid B at 50 μM group which shows no significant effect, the contents of rosmarinic acid and salvianolic acid B declined by approximately 50% and 20% overall compared with the control groups, respectively (Fig. 2F,G).

### Transcription of genes involved in secondary metabolites production are affected by 5-Az

Since the production of tanshinones was increased following the concentration of 5-Az, treatment with 75 μM 5-Az was used for further investigation. Transcription of genes involved in secondary metabolites production in *S. miltiorrhiza* was measured on day 1, 4, 7, and 14 with or without 5-Az treatment.

**Figure 3:**
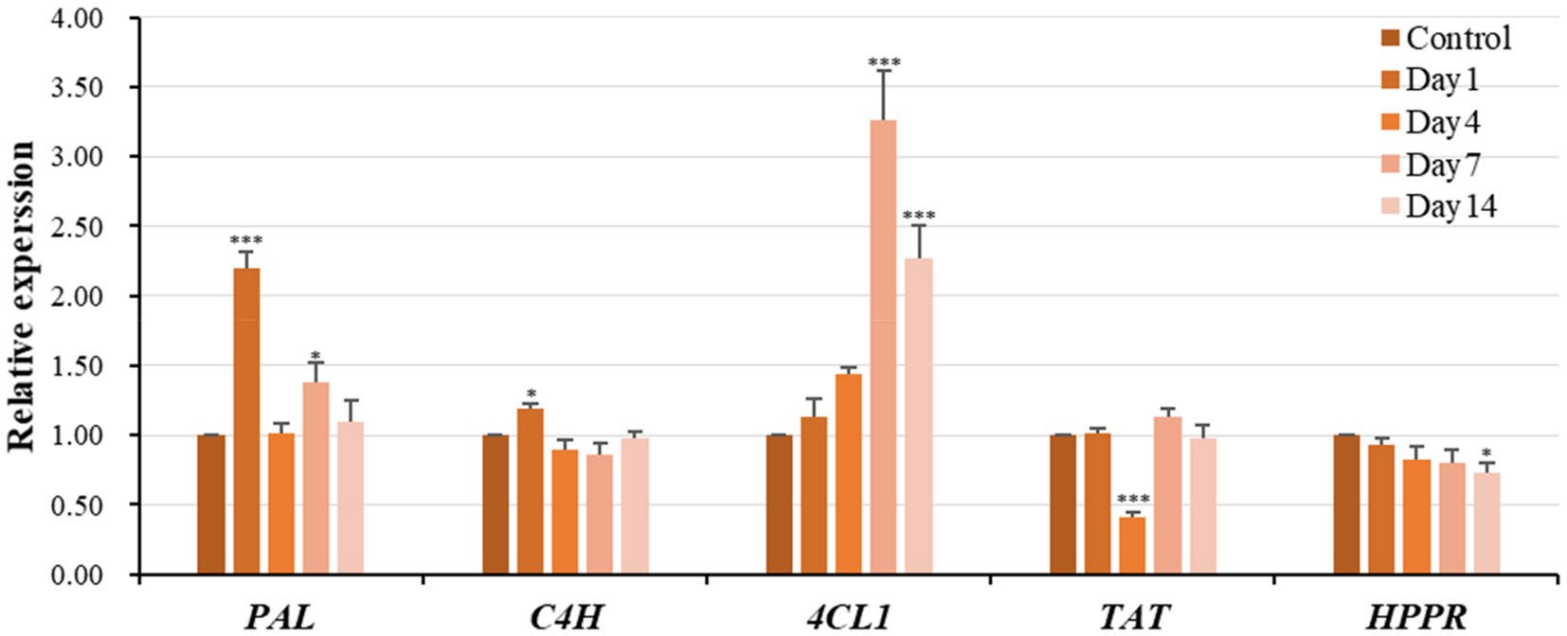
Gene expression of phenolic acid-related compounds in *Salvia miltiorrhiza* hairy roots at 1, 4, 7, and 14 days under 75 μM 5-azacytidine treatment. The values were calculated by using ubiquitin as the endogenous control, following the 2^−ΔΔCt^ method. Bars represent the mean of relative expression ± S.E. of at least three replicates.

The phenylpropanoid pathway and tyrosine-derived pathway are involved in the production of hydrophilic phenolic acids. In the phenylpropanoid pathway, the gene expression of *phenylalanine ammonia-lyase *(*PAL*), *cinnamic acid 4-hydroxylase *(*C4H*) and *4-coumarate:CoA ligase 1* (*4CL1*) were measured during the treatment (Fig. 3). The results showed that the relative expression level of *PAL* was fluctuant within 14 days, the *PAL* expression was significantly increased by 5-Az on day 1 with 119% and day 7 with 38%, but no significant differences on day 4 and day 14. The expression level of *4CL1* showed a notable up-regulation on day 7, and 14 with 220%, 120% respectively. The expression of *C4H* only raised significantly 18% on day 1, but showed no differences at rest of time point. In addition, the expression of *HPPR* and *TAT *on the tyrosine-derived pathway were also measured during the treatment. The results indicated that 5-Az did not modulate the gene expression significantly, except the gene expression level of *TAT* on day 4 with 60% declined and *HPPR* on day 14 with 28% decreased.

**Figure 4:**
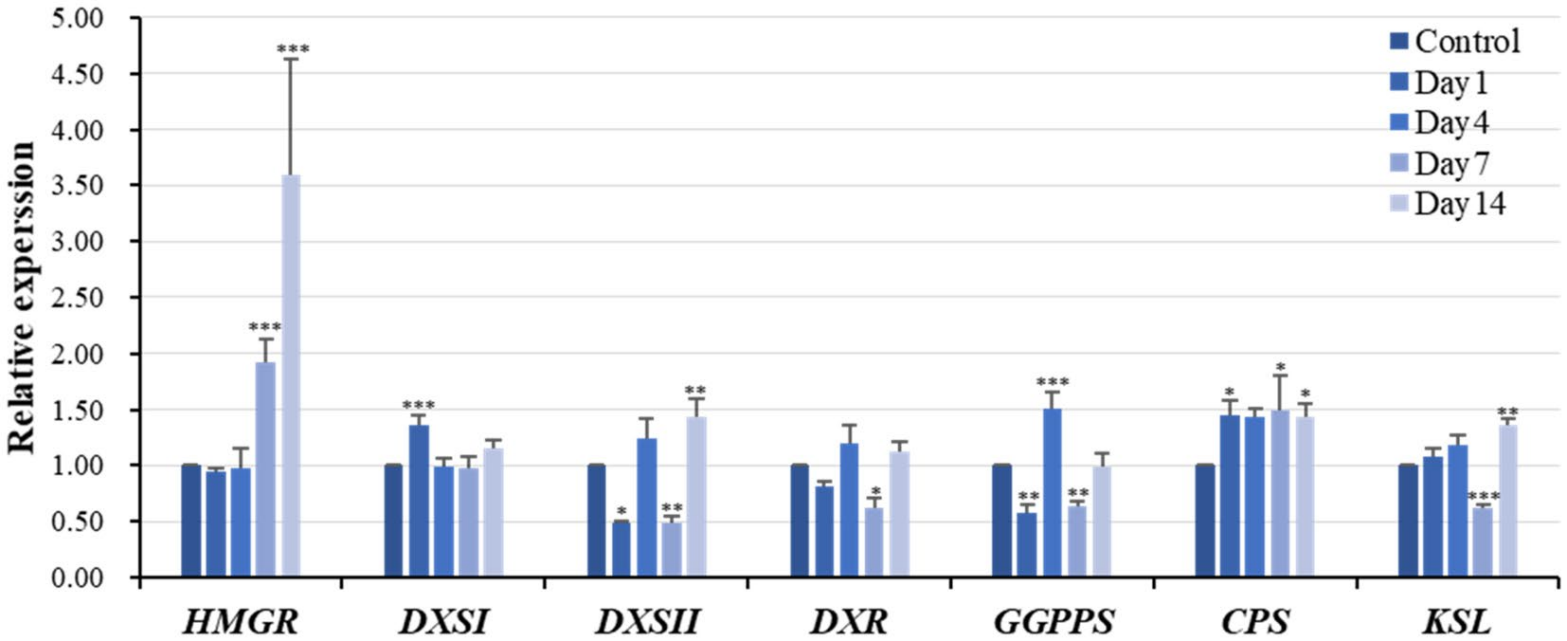
Gene expression of tanshinone-related compounds in *Salvia miltiorrhiza* hairy roots at 1, 4, 7, and 14 days under 75 μM 5-azacytidine treatment. The values were calculated by using ubiquitin as the endogenous control, following the 2^−ΔΔCt^ method. Bars represent the mean of relative expression ± S.E. of at least three replicates.

The mevalonate (MVA) pathway and the methylerythritol phosphate (MEP) pathway are the two biosynthesis pathways that contribute to the accumulation of tanshinones in *S. miltiorrhiza*. As shown in Fig. 4, the level of *hydroxy-3-methylglutaryl-CoA reductase* (*HMGR*) in the MVA pathway was measured during the experiment. There were no significant alternations on day 1 and 4, but the relative gene expression level of *HMGR* increased sharply on day 7 and 14 with 90% and 260%.

In the MEP pathway, the gene expression of *1-deoxy-*d*-xylulose-5-phosphate synthase I* (*DXSI*), *1-deoxy-*d*-xylulose-5-phosphatesynthase II* (*DXSII*), and *1-deoxy-d-xylulose-5-phosphate reductoisomerase* (*DXR*) were measured during treatment. The relative expression of *DXSI* only raised on day 1 around 35%, and showed no differences on day 4, 7 and 14. The gene expression of *DXSII* was fluctuant within 14 days. The gene expression *of DXSII* dropped down significantly around 50% on day 1 and 7, and then up-regulated notably about 40% on day 14. The expression of *DXR* only dropped significantly 40% on day 7, but showed no differences at rest of time points. In addition, genes related to the construction of tanshinone skeleton were also investigated, such as *geranylgeranyl diphosphate synthase* (*GGPPS)*, *CPS* and *KSL. GGPPS* also showed a dramatically changes in the expression level. The relative expression of *GGPPS* decreased 44% on day 1, followed by a significant increase 50% on day 4 and then declined again approximately by 40% on day 7. The gene expression of *KSL* also showed a dramatically changes in the expression level. The relative expression of *KSL* decreased 40% on day 7 and then up-regulated approximately by 40% on day 14. Moreover, the relative expression of *CPS* showed a positive modulation almost at all the time points with approximately 45% up-regulation.

### DNA methylation was changed by 5-Az

**Figure 5:**
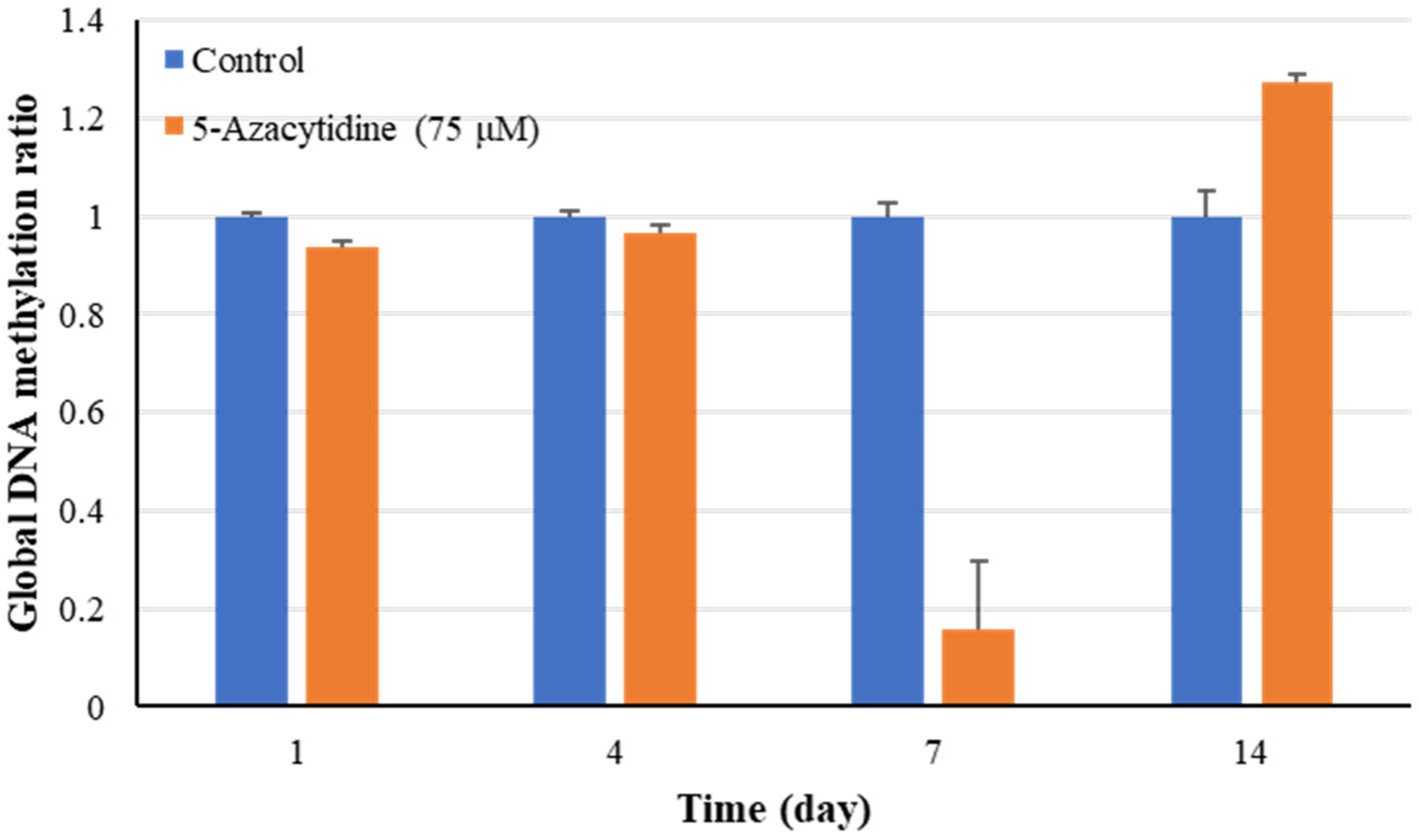
The ratio of global DNA methylation in *Salvia miltiorrhiza* hairy roots at 1, 4, 7, and 14 days with or without 75 μM 5-azacytidine treatment. The values are the mean of at least three replicates ± S.E.

The ratio of methylation levels in genomic DNA was measured on different days with or without 5-Az treatment (Fig. 5). The results indicated that there was no significant difference in the methylation ratio on day 1 and day 4, which was only approximately 5% less than that of the control group. However, the DNA methylation level changed sharply after day 4. The ratio decreased by approximately 84% on day 7, while it increased by 27% compared with the control group on day 14. Therefore, 5-Az indeed exerted its inhibitory activity to influence the methylation ratio in genomic DNA.

### 5-Az alters DNA methylation at potential transcription factor binding sites

**Figure 6:**
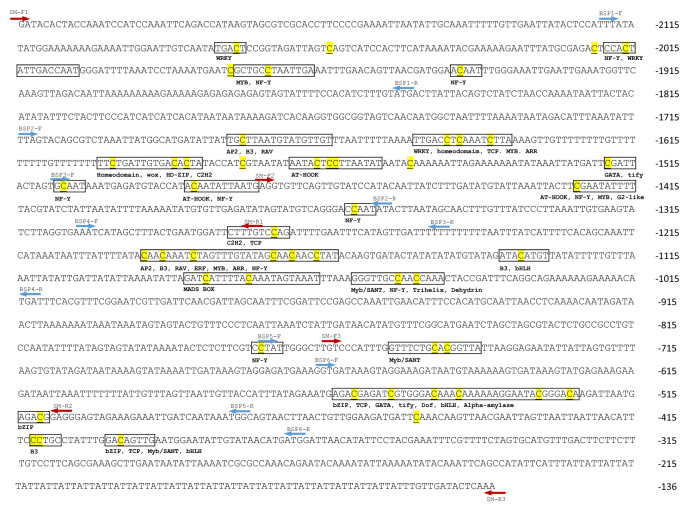
The *S. miltiorrhiza CPS* promoter region was amplified based on the reference from Szymczyk et al. Only the + strand is provided, and the specific primers are underlined. Compared with the control group, the demethylated cytosines are underlined in yellow, and the positions of putative transcription factors binding sites are boxed and indicated below.

Based on the sequence information from Szymczyk et al.^50^, several putative transcription factors (TFs) might bind to specific motifs on the *CPS* promoter and enhance *CPS* gene expression. Meanwhile, since the relative gene expression of *CPS* shown a positive modulation with 40% up-regulation in our experiment, the promoter region of *CPS* has been chosen for further studies. To prove that 5-Az might change the DNA methylation patterns in the promoter region, the methylation level of each cytosine on the *CPS* promotor was investigated. According to our experimental results in the methylation level of genomic DNA, samples were collected on day 7 and processed by bisulfite conversion. Then, specific primers were designed to amplify the promoter region. PCR products were analyzed by next-generation sequencing (NGS), and the results were investigated using PLANTPAN 3.0^51^ for potential TF binding sites (Fig. 6).

Our results indicated that the methylation level of cytosine on the *CPS* promoter was altered during 5-Az treatment compared with the control group. One hundred forty-five cytosines were detected in our amplicons, among which 51 showed decreased methylation levels during 5-Az treatment. The regions of approximately −747 to −511 bp and −1421 to −1412 bp showed a greater alteration in demethylation levels compared with another region; moreover, cytosines at −1412 bp, −745 bp, −560 bp, −554 bp, and −547 bp showed decreased methylation levels to 23.07, 25.01, 40.92, 30.78, and 26.14%, respectively (Table S1).

According to the analysis results, thirty-six different putative TFs binding sites were demethylated, including alpha-amylase, AP2, ARR-B, AT-hook, B3, bet_v_1, bHLH, bZIP, C2H2, C3H, dehydrin, Dof, ERF, G2-like, GATA, GRF, HD-ZIP, homeodomain, LEA_5, MADS box, MIKC, M-type, MYB, MYB/SANT, NAC, NAM, NF-Y, RAV, SBP, TALE, TCP, tify, trihelix, WOX, WRKY, and ZF-HD. Among these TFs, NF-Y and MYB were frequently found at demethylation sites (Table 1).

**Table 1:** Demethylated cytosine sites in the CPS promoter and their corresponding homologous trans-factors from *A. thaliana* and other species.

Trans-factor name	Demethylated cytosine sites
Alpha-amylase	−530
AP2	−1678, -1638, −1194, −1186, −1173, −1170, −1166
ARR-B	−1645, −1643, −1412, −1194, −1191
AT-Hook	−1678, −1566, −1564, −1563, −1486, −1424
B3	−1678, −1486, −1194, −1173, −1170, −1133, −412, −411
bet_v_1	−1645
bHLH	−1412, −1133, −530, −398
bZIP	−2079, −1645, −1507, −1412, −560, −511, −398
C2H2	−1589, −1587, −1273
C3H	−1412, −543
Dehydrin	−1053, −1050
Dof	−1638, −554, −547, −543, −530
ERF	−1638, −1186, −1166
G2-like	−1424
GATA	−1519, −1133, −554
GRF	−1360
HD-ZIP	−1599, −1507
Homeodomain	−2079, −1645, −1599, −1507
LEA_5	−1412
MADS box	−1645, −1184, −1077, −543
MIKC	−543
M-type	−543
MYB	−1980, −1645, −1643, −1424, −1194, −1191, −1173, −1170, −1166
MYB/SANT	−1980, −1645, −1643, −1424, −1412, −1194, −1191, −1173, −1170, −1166, −1053, −747, −745, −398
NAC	−1424, −1412, −547, −543, −530
NAM	−1424, −1412
NF-Y	−2016, −1979, −1943, −1643, −1507, −1486, −1424, −1360, −1191, −1166, −1077, −1053, −1050, −775
RAV	−1678, −1194, −1173, −1170
SBP	−775
TALE	−2079, −1645
TCP	−1943, −1360, −1273, −775, −560, −554, −547, −543, −525, −398
tify	−1519, −1133, −554
Trihelix	−1412, −1133, −1077, −1053
WOX	−1599
WRKY	−2079, −2016, −1645
ZF-HD	−745

## Discussion

Plant secondary metabolites are specific compounds that are produced as part of the defense system and mediators for interactions between plants and environments^52,53^. It has been reported that secondary metabolites have a responsive and regulatory relationship with DNA methylation in plants to adapt to environmental stresses. DNA methylation is recognized as an extensive epigenetic mechanism in plants. Pandey and Pandey-Rai demonstrated that 3-h UV-B treatment could upregulate the DBR2 gene and enhance the concentration of artemisinin up to 1.91-fold in *Artemisia annua*. Moreover, the global level of DNA methylation was reduced after UV-B treatment^15^. This proved that the demethylation of DNA was responsive to UV-B. UV-B radiation also regulated flavonoid biosynthesis through epigenetic mechanisms in *A. annua*. Epigenetic characterization of the *AaPAL1* promotor region for regulation of flavonoid biosynthetic pathway revealed cytosine demethylation at specific sites in *AaMYB1*, *AaMYC* and *AaWRKY* TF binding sites under UV-B treatment^54^. It caused overexpression of the *AaPAL* gene and significantly induced an increase in the total flavonoid content up to 2.4-fold compared to control plants. Similarly, 5-azacytosine (5-AzaC) significantly increased the expression of the gene encoding stilbene synthase 10 (*VaSTS10*) and enhanced resveratrol production in *Vitis amurensis*. The DNA methylation level of the promotor and coding regions of the *VaSTS10* gene was decreased^55^. Based on previous research, changes in DNA methylation can significantly affect secondary metabolite production by regulating the expression of specific genes through their promotors.

In our experiment, 5-Az treatment significantly increased the content of tanshinones in *S. miltiorrhiza* hairy roots and similar trends could also be observed in the gene expression levels. 5-Az treatment not only upregulated the expression of specific genes responsible for precursor of tanshinone synthesis on the MVA pathway, but also the genes related to the formation of tanshinone skeleton. However, the contents of water-soluble compounds, such as rosmarinic acid and salvianolic acid B, were reduced in most of dosages of 5-Az treatment, even though some positive regulations in the phenylpropanoid pathway have been observed. Some reports have indicated that the genes in the tyrosine-derived pathway might play more important roles in the formation of rosmarinic acid and salvianolic acid B^31,56^. The opposite results were reported for water-soluble compounds when experiments were performed using different demethylation reagents, such as 5-AzaC (5-azacytosine), in *S. miltiorrhiza* hairy roots^26^. Their study indicated that the effect of demethylation by 5-AzaC could increase the contents of caffeic acid, rosmarinic acid and salvianolic acid B in *S. miltiorrhiza* hairy roots. Demethylation reagents, such as 5-AzaC, 5-Az, 5-aza-2′-deoxcytidine and 5,6-dihydroazacytidine, have been used to treat host cells to change DNA methylation levels, and their mechanisms are varied^40,57,58^. For example, 5-Az could incorporate into both DNA and RNA, whereas 5-aza-2′-deoxycytidine could only incorporate into DNA. When the DNA methyltransferase encountered the residue of 5-Az or 5-aza-2′-deoxycytidine in DNA, it resulted in an irreversible binding and then losing enzyme activity. These reactions contribute to low methylation level in DNA. Moreover, the incorporated reaction might be different because of the chemical construction. For example, 5,6-dihydroazacytidine was limited incorporation into DNA because of the inefficient phosphorylation by cytidine kinase^59^. Those differences eventually might lead to the different methylation level in the tissue^60^. Moreover, the dosages of DNA methylation inhibitors might play an important role in regulating the biosynthesis of intermediate compounds in plant cells. For example, in the study of *Catharanthus roseus* cell suspension cultures, the concertation of 5-Az was used about 40.95 to 81.9 μM for the formation of secondary metabolites^47^. On the other hand, triterpenoid contents and biosynthetic genes in *Betula platyphylla* were induced by 5-Az with 50–500 μM^49^. In addition, 200 μM 5-Az significantly increased the resveratrol production up to 2.0-fold in *Vitis amurensis*^48^. In conclusion, we believe that different results might arise from the different epigenetic reagents and the concentrations that we used.

The alteration of methylation patterns in promoter might affect the gene expression level and result in the accumulation of secondary metabolites in plants. Yang et al.^26^ indicated that the reduction of methylation levels on the *rosmarinic acid synthase* gene (RAS) promoter could induce gene expression involved in the biosynthesis pathway and then significantly enhance the production of water-soluble compounds in *S. miltiorrhiza* hairy roots. To evaluate the effect of 5-Az on biosynthetic pathway, we observed the gene expression and genomic DNA methylation levels on different days. The results indicated that 5-Az treatment might upregulate the tanshinone biosynthesis pathway and dramatically decrease the global methylation level on day 7. However, an overcompensation of the gene expression level might be happened in the plant cells when the inhibition effect of was alleviated^61^. The level of DNA methylation on day 14 was increased that might be a compensation effect. Therefore, this result implied the decreased methylation level on day 7 might be a transient inhibitory effect of 5-Az in DNA methylation. Thus, a further investigation on promoter region was performed at this time point.

Since 5-Az treatment was able to significantly enhance the expression of *CPS*, which encodes a key enzyme in the tanshinone biosynthesis pathway, we analyzed the methylation profile in the promoter region by NGS. The methylation patterns on the *CPS* promoter were altered by 5-Az, and the methylation level on each cytosine also varied during the treatment. This result indicated the possible relationships between the gene expression of the *CPS* and the demethylation effect of 5-Az. Based on the demonstration by Szymczyk et al., many TF binding sites can be observed in the *CPS* promoter region. We found that 5-Az promoted a demethylation effect on some TF binding sites, and approximately thirty-six different TFs were recognized by PLANTPAN3.0. Among them, MYB and NF-Y were frequently found in our results, as shown in Fig. 6 and Table 1.

TFs modulate gene expression by directly/indirectly binding to DNA sequences, and the numerous TFs in organisms represent vital characteristics in the regulation of cellular processes. One of the largest TF families in plants is the MYB family. Four subfamilies can be classified based on the numbers of adjacent MYB repeats in the DNA binding domain, namely, 1R-MYB, 2R-MYB, 3R-MYB and 4R-MYB. The function of MYB proteins has been studied in various plant species, including *Arabidopsis*, cotton, and rice^62,63^. In previous studies with *S. miltiorrhiza*, MYB proteins have been recognized as a control factor in water-soluble and liposoluble compound production. For example, the overexpression of SmMYB9b can improve the production of tanshinones and related ingredients^20^, and the overexpression of SmMYB2 enhances the production of water-soluble compounds, such as rosmarinic acid and salvianolic acid B^64^. The other common TF found in our results was nuclear factor Y (NF-Y), which is also known as a heme-associated protein/CCAAT box binding factor. NF-Y is composed of three subunits, NF-YA, NF-YB and NF-YC, and these subunits can regulate downstream genes individually or work as a protein complex. Several reports have found that NF-Y responds to embryogenesis, flowering time and abiotic stress in *A. thaliana*^65,66^. Based on our findings, especially of the demethylation effect on MYB and NF-Y binding motifs on the *CPS* promoter, further important investigations might be inspired. For instance, whether the demethylation effect of 5-Az could alter the affinity between TFs and their binding sites? whether the demethylation effect of 5-Az could modulate TF promoters? or whether the interaction between TFs and promoters under 5-Az treatment are worthy of being investigated.

We believe that the function of 5-Az in *S. miltiorrhiza* hairy roots might alter DNA methylation patterns and cause changes in gene expression related to secondary metabolism; for example, tanshinone production was improved. In our study, the *CPS* promoter region was used as a target as we tried to find a linkage between the demethylation effect of 5-Az and tanshinone production. We found that some DNA sequence motifs tend to be demethylated during 5-Az treatment, but whether the demethylation effect of these DNA sequence motifs affects the affinity between DNA and TFs is still debatable. Regardless of whether the demethylation effect of the DNA sequence motifs on the *CPS* promoter was the cause or a consequence, our study provides evidence for the possibility of a novel metabolic engineering strategy to promote important secondary metabolite production in medicinal plants.

## Materials and methods

### Chemicals and reagents

Gamborgs B-5 basal medium, sucrose, 5-azacytidine and tanshinone IIA were purchased from Sigma-Aldrich (St. Louis, MO, USA). Tanshinone I, dihydrotanshinone I, and salvianolic acid B were purchased from Wuhan ChemFaces Biochemical Co., Ltd. (WUHAN, HUBEI, China). Rosmarinic acid was purchased from ChromaDex (Los Angeles, CA, USA). Cryptotanshinone was purchased from Tokyo Chemical Industry Co., Ltd. (Tokyo, Japan). HPLC grade ethanol was purchased from Honeywell Chemicals (Seelze, Germany). HPLC grade acetonitrile was purchased from Fisher Scientific (Seoul, Korea).

### Hairy roots culture

*S. miltiorrhiza* cultivars were purchased from Taiwan Agricultural Research Institute Council of Agriculture, Executive Yuan, Taichung, Taiwan, and its hairy roots were induced from leaf explants by *A. rhizogenes* (LBA1334) infection^67^. Hairy roots were maintained in phytohormone-free (0.5 ×) Gamborgs B5 liquid medium with 3% sucrose in the dark (pH 5.2 ± 0.1) at 25 ± 1 °C and subcultured every 4 weeks. All plant materials used in this study were complied with local and national regulations.

### 5-Azacytidine treatment

All experiments were carried out in a 125 mL flask inoculated with 0.2 g fresh *S. miltiorrhiza* hairy roots. Experimental treatments were processed by diluting 5-Az stock solution to different concentrations (12.5 μM, 25 μM, 50 μM and 75 μM) in (0.5 ×) Gamborgs B5 liquid medium with 3% sucrose. A group without any treatment was used as a control. The hairy roots were harvested after 1, 4, 7, and 14 days for qRT-PCR and global DNA methylation assays; 28 days for HPLC analysis; and 7 days for next-generation sequencing.

### Extraction and high-performance liquid chromatography (HPLC) analysis

The hairy roots samples were harvested and weighted after 28 days of treatment and then processed using a FreeZone 6 Freeze Dry System (Labconco, USA) until reaching a constant dry weight. Samples were ground into powder and extracted with 80% ethanol (0.05 mL mg^−1^) in an ultrasonic bath for 50 min. The extracts were filtered through a 0.45 μm PVDF membrane filter before HPLC analysis.

Chromatography analysis was performed with a Fortis C18 column (particle size 5 μm, 250 × 4.6 mm; Fortis, UK) connected to a photodiode array (PDA)-equipped Shimadzu 10AP HPLC System (Shimadzu, Japan). The flow rate was 1 mL min^−1^, and the temperature was set at 30 °C. Acetonitrile (A) and 0.5% aqueous acetic acid (v/v) (B) were used as the mobile phases. Gradient elution was performed with a linear gradient according to the following program: t = 0 min, 98% A; t = 80 min, 10%. The sample injection volume was 10 μL and different detection wavelengths were selected to compare its UV absorption and retention time for the identification of individual compounds. The detection wavelengths were 254 nm for tanshinone I and dihydrotanshinone I; 270 nm for salvianolic acid B, cryptotanshinone and tanshinone IIA; and 330 nm for rosmarinic acid.

### RNA isolation and qRT-PCR

The expression of tanshinone biosynthesis genes, including genes encoding 3-hydroxy-3-methylglutaryl-CoA reductase (HMGR), 1-deoxy-d-xylulose-5-phosphate synthase I (DXSI), 1-deoxy-d-xylulose-5-phosphatesynthase II (DXSII), 1-deoxy-d-xylulose-5-phosphate reductoisomerase (DXR), geranylgeranyl diphosphate synthase (GGPPS), copalyl diphosphate synthase (CPS), kaurene synthase-like (KSL), as well as phenolic acid biosynthesis genes, including those encoding phenylalanine ammonia-lyase (PAL), cinnamic acid 4-hydroxylase (C4H), 4-coumarate:CoA ligase 1 (4CL1), tyrosine aminotransferase (TAT), and 4-hydroxyphenylpyruvate reductase (HPPR), were investigated by quantitative real-time PCR in the hairy roots of *S. miltiorrhiza*. Ubiquitin was used as the reference gene^68^. The specific primers were obtained from published articles^69,70^. Total RNA was extracted using a PureLink™ RNA Mini Kit (Invitrogen, USA), and cDNA was prepared with superscript IV VILO master mix (Invitrogen, USA) according to the manufacturer’s instructions. Real-time quantitative PCR (qRT-PCR) was performed with 100 nM each of the forward and reverse primers, powerup SYBR green master mix (Applied Biosystems, USA), and 20 ng of cDNA template in a total volume of 10 μL. The amplification program was as follows: 50 °C for 2 min, 95 °C for 2 min, and then 40 cycles of 95 °C for 15 s and 60 °C for 1 min. The relative expression of genes was calculated by using ubiquitin as an endogenous control following the 2^−ΔΔCt^ (cycle threshold) method^71^.

### DNA isolation

Total DNA was extracted using a PureLink™ Plant total DNA purification kit (Invitrogen, USA) according to the manufacturer’s instructions. Samples were extracted after 1, 4, 7, and 14 days of treatment in 75 µM 5-Az, and the samples without treatment were used as the control group. The DNA quality was measured based on the A260/280 and A260/230 parameters using NanoVue (GE Healthcare).

### PCR

Based on the results of Szymczyk et al., specific primers were designed to construct the *CPS* promoter and are listed in Table 2. The PCR mixture contained 1 U of DNA polymerase (KAPA), 0.2 mM dNTPs, and 2 mM MgCl_2_ in the reaction buffer. Ten nanograms of fresh DNA was used as template and the reaction was performed in a volume of 25 µL. The primer concentration was 0.4 µM. The PCR parameters were as follows: initial denaturation at 98 °C for 1 min; 40 cycles of denaturation at 98 °C for 1 min, primer annealing at 51 °C for 1 min, and extension at 72 °C for 40 s, and final extension at 72 °C for 3 min.

**Table 2:** Primers used in this study.

**Bisulfite primer (5′–3′)**	
BSP1-F	TTTATATATGGAAAAAAAGAAAATTGGAATTG
BSP1-R	TCATACAAAAATATAAAAAATACTACTCTCTCTC
BSP2-F	TTTAGTATAGYGTTTAAATTATGGTATGATATTATTG
BSP2-R	TCAAAAATAATTATATAAATACAACTAAACACC
BSP3-F	GTAATAAATGAGATGTATTATATAATATTAATGAGG
BSP3-R	AAAAAATCAACTATAAAATTCAAAATCTAAAC
BSP4-F	AATTATAGTTTTATTGAATGGATTTTTTGTTTAG
BSP4-R	ATCATATTTTTCTTTTTTCTACCTAAAATC
BSP5-F	TATTGGGTTTGTTTTATTTGGTTTTTG
BSP5-R	ACCATTTATTAATCAATTTCTTTCTAC
BSP6-F	GAGATYGTGGGATAAATAAAAAAGG
BSP6-R	TATATTAATCCATCATATTATACAATATTCCATTCAAC
**PCR primer (5′–3′)**	
SM-F1	TCATAGAGAGAGTTGTCG
SM-R1	GGACAAAGAATCCATTCAG
SM-F2	GAGGTGTTCAGTTGTATC
SM-R2	CCTCCGTCTCATTAATCT
SM-F3	TGTCCCATTTGGTTTCTG
SM-R3	CGGGTTTAGGATTTGAGTT

### Measurement of DNA methylation levels

The level of DNA methylation was measured by a global DNA methylation assay kit (Invitrogen, USA) (ab233486) according to the manufacturer’s instructions, and 50 ng of DNA was used in each reaction. In this assay, DNA was bound to strip wells with high DNA affinity, and the methyl group on the cytosine was detected by using antibodies. The level of methylation was quantified calorimetrically using the absorbance at 450 nm. A standard curve was generated from a range of 0.1–5% of the total methylation level, and the equation was generated using a second-order polynomial curve.

### Bisulfite conversion

The genomic DNA were extracted after 1, 4, 7, and 14 days of treatment in 75 µM 5-Az, and the samples without treatment were used as the control group. The genomic DNA samples from the control group and 5-Az-treated hairy roots were converted by a fast bisulfite conversion kit (Invitrogen, USA) (ab117127) according to the manufacturer’s recommendations. Bisulfite conversion was used as a tool to detect the methylation level in the DNA fragments due to specific changes in the unmethylated cytosine. Cytosine without methyl groups was converted into uracil, whereas methylated cytosine was unaffected.

### Next-generation sequencing

To understand the methylation level of each cytosine in the CPS promoter, next-generation sequencing was performed. Bisulfite-converted DNA was used as the DNA template and amplified with the primers listed in Table 2. The conditions for PCR were described above, but the annealing temperature was changed to 50 °C. PCR products were mixed in equal volume and ligated with adaptors. Afterwards, the mixture of PCR products was analyzed by Illumina MiSeq System sequencing, and the results were compared to the original *CPS* promoter as a reference gene. The methylation level of each cytosine was calculated by CLC Genomics Workbench v10.

### Data analysis

All the experiences were performed independently in triplicate, and the data presented as the means ± standard errors of the mean (SEM). To evaluate the significance of results, statistical analysis was using one-way analysis of variance (ANOVA) with Fisher's least significant difference (LSD) method for the post-hoc analysis. Differences with P < 0.05 were considered significant.

## Supplementary Information

Supplementary Information.
