# Supplementary material for: 5-Azacytidine increases tanshinone production in Salvia miltiorrhiza hairy roots through epigenetic modulation
Source: Sci Rep. 2022 Jun 7;12:9349. doi: 10.1038/s41598-022-12577-8 (PMC9174287; doi:10.1038/s41598-022-12577-8)
Supplement: Supplementary file 1 — Supplementary Information. [file 41598_2022_12577_MOESM1_ESM.pdf]

Supplementary material

Table S1. Demethylation level of each cytosine site in CPS promoter region were analyzed by NGS. Samples were treated with 75 µM 5-Az, and the samples without treatment were used as the control group. gDNA were extracted on day 7, and processed by bisulfite conversion. PCR conditions were described in materials and methods section.

Cytosine site	Decreasing Level of methylation (%)
-2079	6.48%
-2064	3.93%
-2021	2.66%
-2016	6.18%
-1980	1.04%
-1974	5.88%
-1943	10.84%
-1678	2.40%
-1645	1.89%
-1643	0.57%
-1638	6.50%
-1599	2.83%
-1589	2.94%
-1587	2.98%
-1578	2.54%
-1566	1.48%
-1564	0.95%
-1563	2.53%
-1551	4.00%
-1519	0.59%
-1507	1.19%
-1486	3.11%

Cytosine site	Decreasing Level of methylation (%)
-1424	16.97%
-1412	23.07%
-1360	3.26%
-1273	0.03%
-1194	0.50%
-1191	4.94%
-1186	0.52%
-1173	1.07%
-1170	1.66%
-1166	1.92%
-1133	2.01%
-1084	0.92%
-1077	0.19%
-1053	1.51%
-1050	0.12%
-775	0.49%
-747	12.85%
-745	25.01%
-560	40.92%
-554	30.78%
-547	26.14%
-543	14.03%

Cytosine site	Decreasing Level of methylation (%)
-530	17.84%
-525	16.24%
-511	18.87%
-450	1.92%
-412	1.45%
-411	0.81%
-398	3.73%

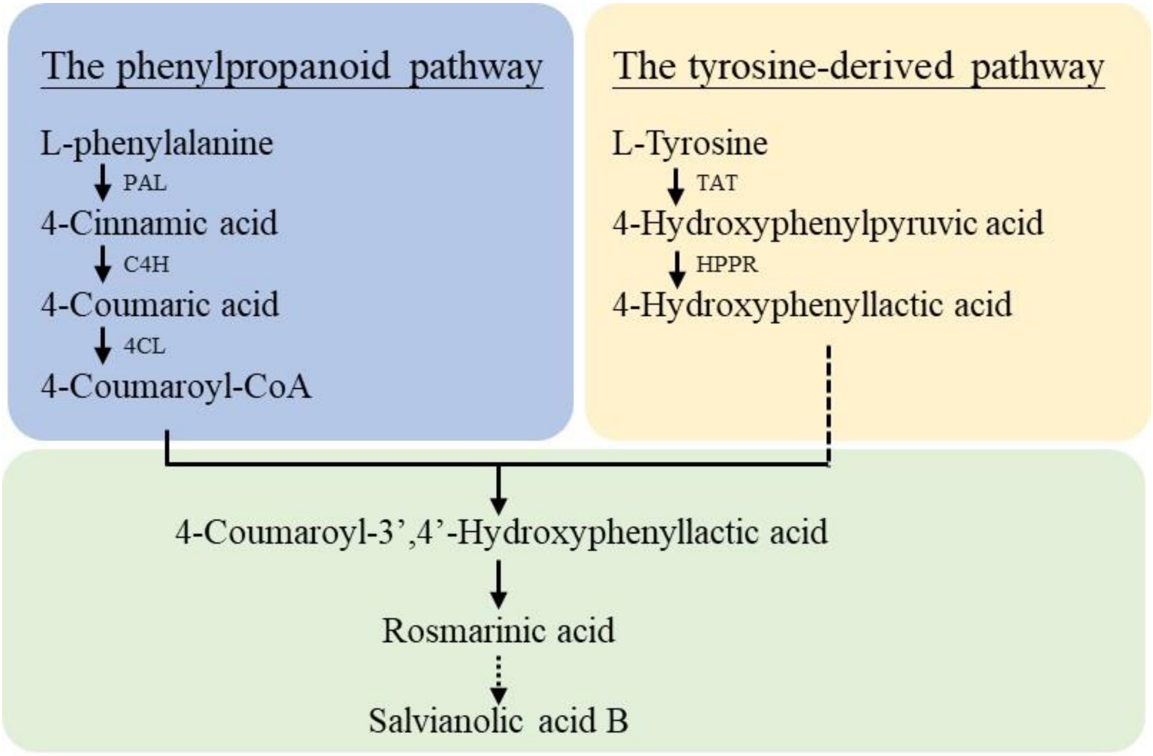

Figure S1. The biosynthetic pathway leading to rosmarinic acid and salvianolic acid B in S. miltiorrhiza hairy roots. TAT: tyrosine aminotransferase; HPPR: 4-hydroxyphenylpyruvate reductase; PAL: phenylalanine ammonia-lyase; C4H: cinnamic acid 4-hydroxylase; 4CL: 4-coumarate-CoA ligase; hydroxylase. Solid line: the verified biosynthesis process; dotted line: proposed biosynthesis processes.

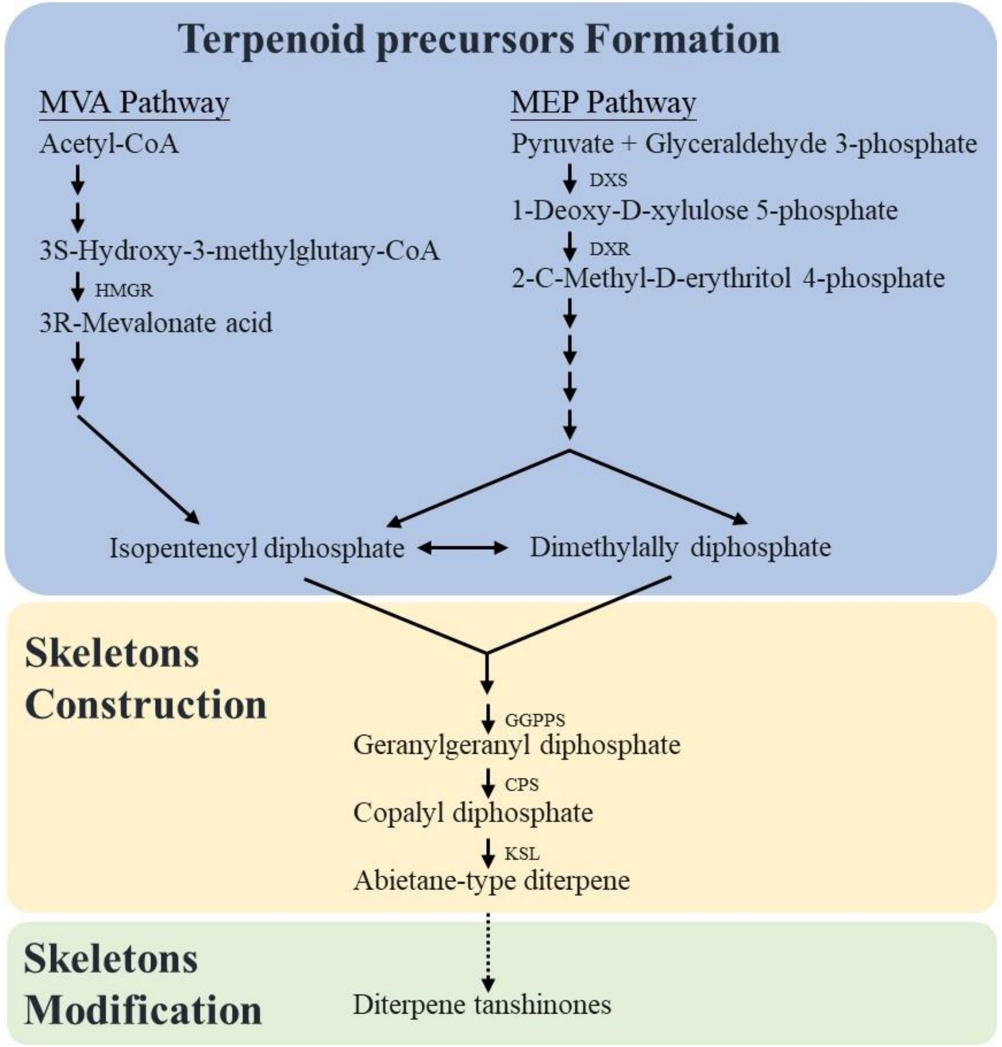

Figure S2. The biosynthetic pathway leading to tanshinones in *S. miltiorrhiza* hairy roots. HMGR: hydroxy-3-methylglutaryl-CoA reductase ;DXSI: 1-deoxy-D-xylulose-5-phosphate synthase I; DXSII: 1-deoxy-D-xylulose-5-phosphatesynthase II; DXR: 1-deoxy-D-xylulose-5-phosphate reductoisomerase; GGPPS: geranylgeranyl diphosphate synthase; CPS: copalyl diphosphate synthase; KSL: kaurene synthase-like. Solid line: the verified biosynthesis process; dotted line: proposed biosynthesis processes.

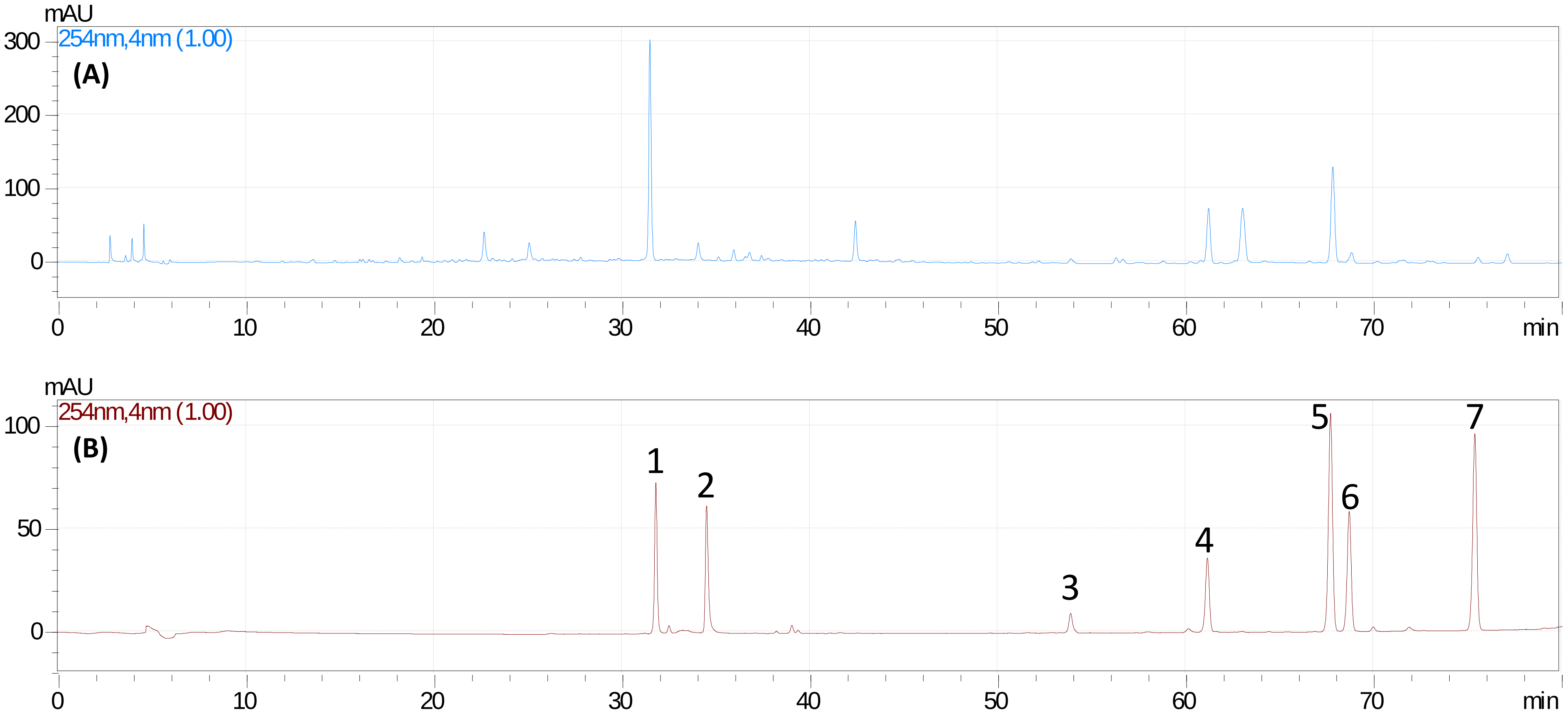

Figure S3. HPLC chromatograms (UV chromatogram at 254 nm) of *S. miltiorrhiza* hairy roots (A), and 7 reference standards in this study (B). (B):1, rosmarinic acid; 2, salvianolic acid B; 3, tanshinone IIB; 4, dihydrotanshinone I; 5, cryptotanshinone; 6, tanshinone I; 7, Tanshinone IIA.
